# Multidisciplinary management of disseminated tuberculosis with tuberculous meningitis and active hepatitis B in a critically ill pregnant woman in the third trimester: a case report

**DOI:** 10.1186/s40249-026-01519-5

**Published:** 2026-11-10

**Authors:** Ju Zhao, Liming Zhang, Yun Wang, Chengjin Zhao, Guodong Cheng

**Affiliations:** The Fourth People’s Hospital of Qinghai, Xining, 810000 China

**Keywords:** Case report, Disseminated tuberculosis, Hepatitis B, Multidisciplinary management, Pregnant woman

## Abstract

**Background:**

Tuberculous meningitis (TBM) in pregnancy is rare but life-threatening, with substantial maternal and fetal risks. Third-trimester disseminated tuberculosis involving the central nervous system with concurrent active hepatitis B infection has scarcely been reported, particularly among remote pastoral populations with limited healthcare access. This report highlights the diagnostic and therapeutic challenges of this triple comorbidity and underscores the value of multidisciplinary team (MDT) management in such complex scenarios.

**Case presentation:**

A 24-year-old pastoralist at 30 weeks of gestation presented with headache, vomiting, fever, and nuchal rigidity. Cerebrospinal fluidanalysis confirmed TBM, and chest computed tomography revealed diffuse miliary nodules suggestive of hematogenous disseminated tuberculosis. Serology was positive for hepatitis B surface antigen, e-antigen, and core antibody, indicating active viral replication. Given life-threatening intracranial hypertension [intracranial pressure (ICP) > 330 mmH_2_O] in late pregnancy, osmotic agents were withheld for fetal safety, and lumbar cisternal drainage was performed. An MDT comprising respiratory medicine, obstetrics, hepatology, pharmacy, and critical care nursing specialties was assembled. Anti-tuberculosis therapy was intensified to isoniazid, rifampicin, linezolid, and moxifloxacin; ethambutol was discontinued due to fetal optic neuritis, and tenofovir was added for hepatitis B management. Following stabilization, the patient was transferred to a higher-level facility at 35 weeks of gestation and underwent cesarean section at 36 weeks, delivering a live preterm female infant (2320 g, 1-min Apgar score of 8). Both mother and neonate were discharged without complications on December 1, 2025.

**Conclusions:**

This case demonstrates that early recognition, individualized anti-tuberculosis regimens, and coordinated multidisciplinary collaboration are critical for achieving favorable outcomes in pregnant patients with disseminated tuberculosis complicated by TBM and concurrent hepatitis B infection. The successful management relied on integrated critical care and a multidisciplinary approach, which proved effective even in resource-limited pastoral settings.

**Graphical Abstract:**

**Figure:**
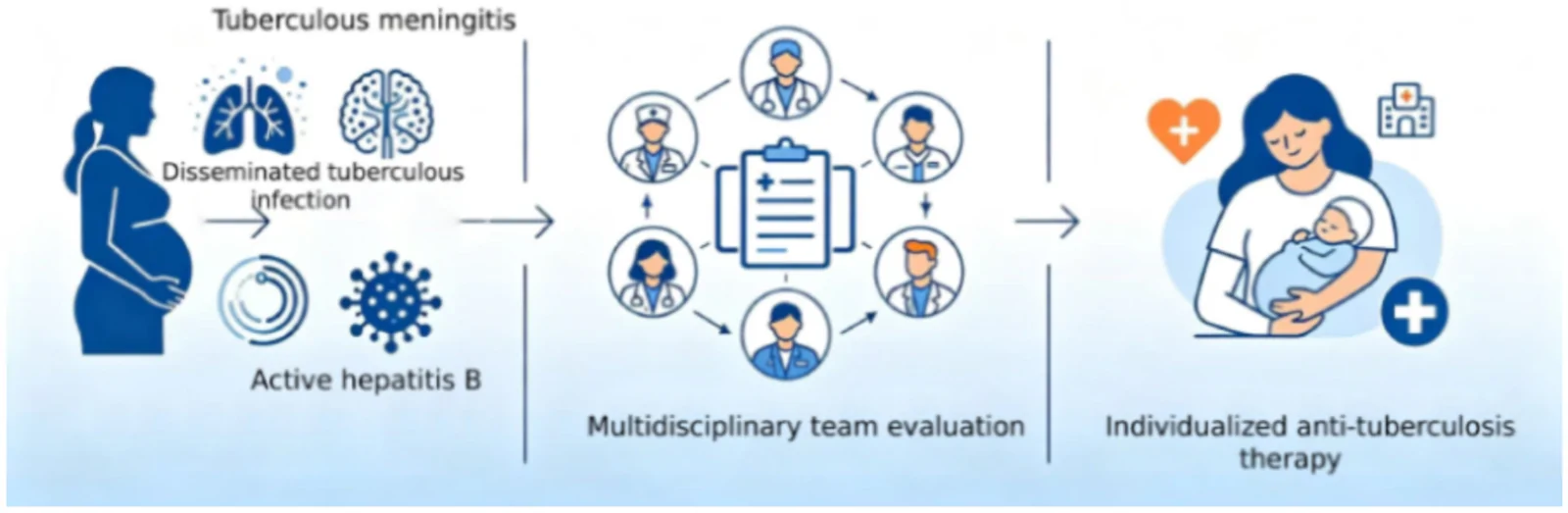

## Background

Tuberculosis (TB) is a chronic infectious disease caused by *Mycobacterium tuberculosis* (MTB) and is primarily transmitted through the air via droplet nuclei. It remains a major global public health concern and one of the leading causes of death from infectious diseases worldwide [1]. During pregnancy, elevated levels of estrogen and progesterone suppress T-lymphocyte function, thereby increasing the risk of tuberculosis. Tuberculosis during pregnancy may result from a new acquired infection or from the progression or reactivation of pre-existing, untreated tuberculosis [2, 3]. Although the specific mechanisms by which pregnancy affects tuberculosis risk remain unclear, studies indicate that pregnancy may increase the risk of active tuberculosis by two- to threefold [4]. Another perspective holds that pregnancy does not directly increase susceptibility to tuberculosis nor necessarily alter disease progression. The key reason lies in the unique physiological changes of pregnancy, such as neuroendocrine regulation and relative immunosuppression, which may mask or confound typical tuberculosis symptoms, making them difficult to detect. Concurrently, the nonspecific discomforts common to pregnancy can overlap with early tuberculosis manifestations, often leading to delayed diagnosis and increasing the likelihood of progression from latent to active disease [5–7].

The true global prevalence of tuberculosis in pregnancy remains unkown. Because many affected women are asymptomatic or lack typical symptoms, a substantial proportion of pregnant women with active tuberculosis may go undetected. Furthermore, given the potential risks of radiological imaging tests to the fetus, diagnosis and treatment during pregnancy are frequently delayed. For tuberculosis during pregnancy or the perinatal period, a high index of clinical suspicion, together with early targeted investigation and standardized diagnosis and treatment, is a key step in improving outcomes in this high-risk population [8, 10]. According to the WHO Global Tuberculosis Report 2025, an estimated 10.7 million new TB cases and over 1.2 million deaths occurred globally in 2024, representing a 2% decline in incidence and 3% reduction in deaths compared with 2023. China accounts for 6.5% of global cases, ranking fourth after India (25%), Indonesia (10%), and the Philippines (6.8%) [9]. Tuberculous meningitis (TBM) is the most severe clinical form of tuberculosis, occurring when MTB disseminates from the lungs to the brain. The disease typically has an insidious onset and slow progression, characterized by persistent headaches lasting days to weeks, neck stiffness, and fever. Early diagnosis and prompt treatment before the onset of coma can significantly reduce mortality and disability [11].

Early diagnosis of tuberculosis during pregnancy is critically important; however, the optimal diagnostic strategy and effective treatment regimen remain to be established. Reports of the concurrent presence of hematogenous disseminated pulmonary tuberculosis, TBM, and hepatitis B with " triple-positive" serology (i.e., “big three positive,” positive for HBsAg, HBeAg, and anti-HBc) are exceedingly rare, and the clinical management of such complex cases in late pregnancy poses substantial challenges. In this case report, we describe the diagnostic and therapeutic course of a 24-year-old herder from a pastoral community in Qinghai who presented with in her third trimester with a triad of conditions. We detail the multidisciplinary management process, encompassing diagnostic workup (lumbar puncture, cerebrospinal fluid analysis, and imaging), therapeutic decision workup (selection and adjustment of anti-tuberculosis regimens, and antiviral therapy for hepatitis B), and perinatal care coordination. The objectives of this report are threefold: (1) to underscore the diagnostic and therapeutic complexities in this understudied population; (2) to illustrate the utility of MDT collaboration in the care of critically ill pregnant patients with coexisting infections; and (3) to offer clinical insights for improving tuberculosis care in remote pastoral settings with constrained healthcare resources. To our knowledge, the simultaneous occurrence of these three conditions in a pregnant woman from a nomadic pastoral community with limited healthcare access has rarely been reported. We therefore present our diagnostic approaches, therapeutic interventions, and clinical outcomes, followed by a discussion of their implications for clinical practice and future research.

## Case presentation

### Patient demographics and initial presentation

A 24-year-old herder from a pastoral community in Qinghai, gravida 1 para 0, at 30 weeks of gestation, presented with a 2-week history of intermittent headache and vomiting, accompanied by worsening low back pain for 1 week. She reported a history of close contact with a sister who had been diagnosed with pulmonary tuberculosis. On admission, she was drowsy with reduced alertness. Her vital signs were as follows: axillary temperature 38.0 °C, heart rate 90 beats/min, respiratory rate 25 breaths/min, blood pressure 125/75 mmHg, and peripheral oxygen saturation of 97% while receiving supplemental oxygen at 2 L/min via nasal cannula. Physical examination revealed nuchal rigidity, positive Kernig's and Brudzinski's signs, and conjunctival injection in the right eye. No skin rash, jaundice, or peripheral lymphadenopathy was observed. Chest auscultation revealed coarse breath sounds bilaterally, without dry or moist rales or pleural friction rub.

Initial evaluation at the referring hospital (October 6–10, 2025).

**Fig. 1:**
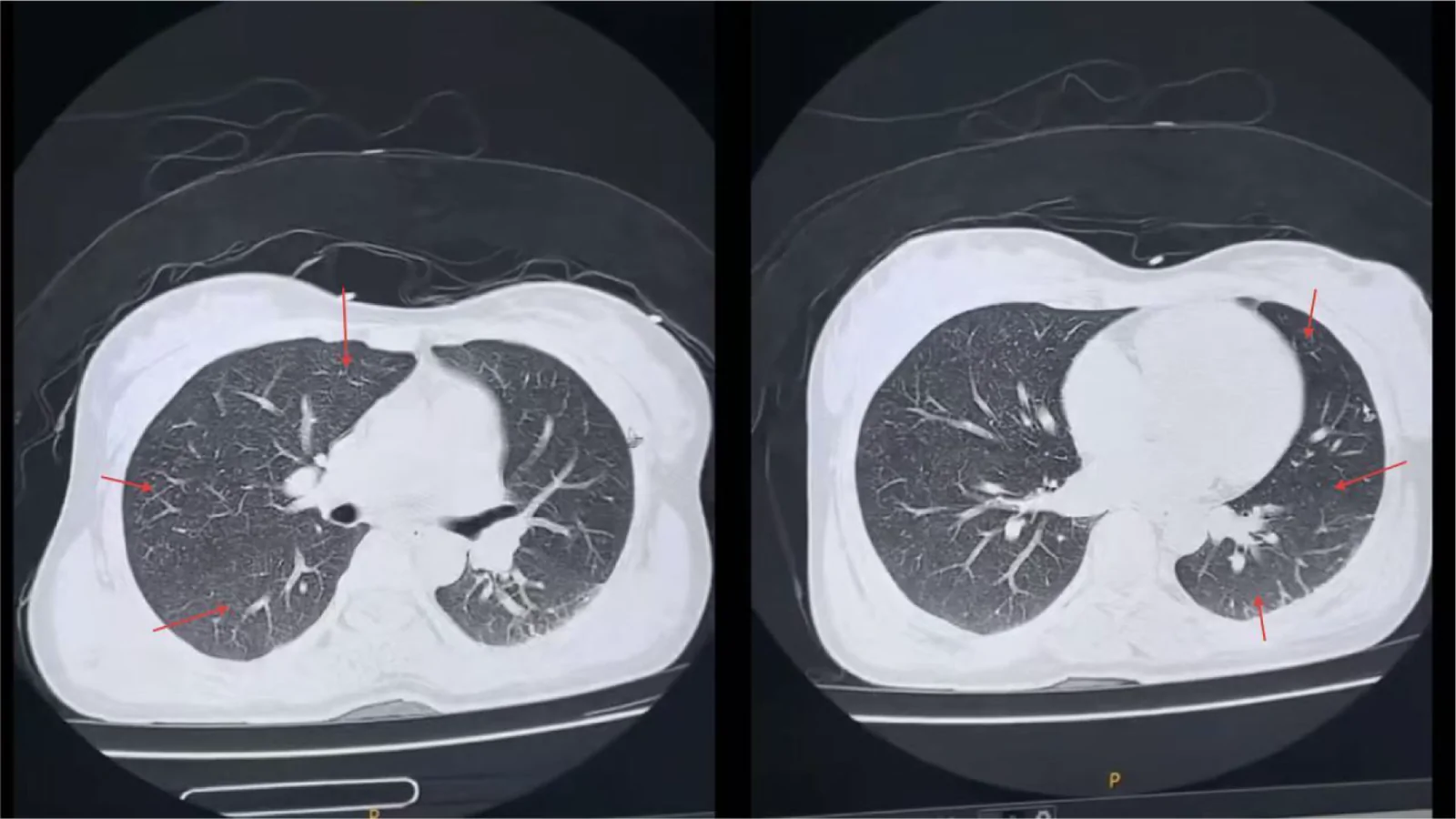
Chest computed tomography image of the critically ill pregnant patient with military tuberculosis. Arrows indicate diffuse military nodules distributed throughout both lung fields

The patient initially presented to Qinghai Red Cross Hospital on October 6, 2025, with a chief complaint of low back pain for 3 days. Urinary ultrasound revealed severe hydronephrosis of the right renal pelvis, and a right nephrostomy was performed after urological consultation, resulting in marked relief of back pain. She subsequently developed persistent headache. Lumbar puncture performed on October 10 demonstrated an opening pressure exceeding 330 mmH_2_O. CSF analysis showed a nucleated cell count of 130 × 10^6^/L, protein 2.37 g/L, glucose 0.59 mmol/L, and markedly decreased chloride—findings consistent with TBM. Serial lumbar punctures were performed during subsequent treatment for monitoring, and the changes in CSF routine and biochemical parameters are shown in Table 1. CSF Xpert MTB/RIF assay detected trace amounts of MTB, and rifampicin resistance was not confirmed. Chest computed tomography (CT) revealed diffuse miliary nodules in both lungs, confirming hematogenous disseminated pulmonary tuberculosis (Fig. 1). Serological testing was positive for hepatitis B surface antigen, hepatitis B e-antigen, and hepatitis B core antibody (triple-positive), with a hepatitis B virus (HBV) DNA load of 1.94 × 10^8^ IU/ml, indicating active viral replication. The tuberculin skin test and interferon-gamma release assay (IGRA) were both positive (N value 0.04 IU/ml; T-N/P-N ratio 2.08). HIV testing was negative.

**Table 1:** Dynamic changes in cerebrospinal fluid parameters during treatment in a pregnant patient with military tuberculosis and tuberculous meningitis

Parameter	10 Oct 2025	13 Oct 2025	16 Oct 2025	19 Oct 2025	21 Oct 2025	23 Oct 2025	14 Nov 2025
Routine analysis							
Color	Colorless	Colorless	Light yellow	Light yellow	Light yellow	Light yellow	Light yellow
Turbidity	Clear	Clear	Clear	Clear	Clear	Clear	Clear
Clot	None	None	None	None	None	None	None
P-Qual	+++	+++	+++	+++	++	++	+
TNC (× 10^6^/L)	130	98	180	**380**	**160**	**72**	**60**
Mono (%)	90	30	30	40	40	45	20
Poly (%)	10	70	70	60	60	55	80
Biochemical Analysis							
K⁺, mmol/L	2.22	2.97	2.26	2.46	3.20	3.03	2.68
Na⁺, mmol/L	124.0	136.4	137.0	134.7	139.1	143.9	138.4
Cl⁻, mmol/L	104.8	116.0	115.9	115.8	112.9	119.4	116.7
GLU, mmol/L	0.59	0.77	0.52	0.49	1.16	0.37	1.11
LDH, U/L	190	140	121	185	86	94	54
ADA, U/L	5.32	10.43	10.76	8.68	7.58	8.15	5.97
CSF TP, g/L	2.37	2.70	2.58	2.57	1.78	1.61	1.44

Bold formatting is used to highlight the key parameters that showed significant changes or abnormal values during the treatment period

The reference ranges are approximate and may vary between laboratories. Bold data highlights key parameters with significant changes or turning points compared to the previous test. *P-Qual* Protein qualitative, *TNC* Total nucleated cell count, *Mono* Mononuclear cells, *Poly* Polymorphonuclear cells, *CSF* cerebrospinal fluid, *LDH* Lactate Dehydrogenase, *ADA* Adenosine Deaminase

### Intensive care management at our hospital (October 10–11, 2025)

Upon transfer to the Department of Critical Care Medicine, urgent multidisciplinary consultations were obtained involving respiratory medicine, obstetrics and gynecology, hepatology, endocrinology, and clinical pharmacy. Given life-threatening intracranial hypertension in the setting of late pregnancy, osmotic agents (mannitol and glycerol fructose) were withheld due to fetal safety concerns (FDA Pregnancy Category C), and lumbar cisternal CSF drainage was performed to control intracranial pressure.

Arterial blood gas analysis revealed pH 7.49, partial pressure of carbon dioxide (PaCO_2_) 24 mmHg, partial pressure of oxygen (PaO_2_) 95 mmHg, Na⁺ 123 mmol/L, K⁺ 3.2 mmol/L, Ca^2^⁺ 1.07 mmol/L, glucose 7.4 mmol/L, and lactate 1.4 mmol/L. Laboratory findings included a white blood cell count of 8.05 × 10^9^/L with 86.20% neutrophils, hemoglobin 98 g/L, and procalcitonin 0.08 ng/ml.

On October 10, an initial anti-tuberculosis regimen comprising isoniazid (0.6 g once daily), rifampicin (0.45 g once daily), and ethambutol (0.75 g once daily) was initiated. Pyrazinamide was omitted because of severe gastrointestinal intolerance, and this regimen was continued until October 15. Furosemide (20 mg every 12 h) was administered intravenously to maintain fluid balance; however, after 5 days of treatment, ultrasonography revealed decreased amniotic fluid volume, and furosemide was consequently discontinued on October 15. Throughout this period, electrolytes were closely monitored, and hyponatremia and hypokalemia were actively corrected.

### Management of comorbidities and treatment adjustment (October 11–15, 2025)

The patient's temperature peaked at 38.7 °C. Etiological workup revealed positive anti-*Mycoplasma pneumoniae* IgM, and sputum culture grew *Candida albicans*. Accordingly, oral azithromycin (250 mg once daily) was administered for 5 days (October 11–15), and nystatin mouthwash was applied topically for oral candidiasis. Thyroid function tests showed a thyroid-stimulating hormone (TSH) level of 5.022 mU/L with normal free thyroxine (FT4), consistent with subclinical hypothyroidism of pregnancy; levothyroxine (25 μg once daily) was initiated on October 12, with normalization of thyroid function on subsequent testing. Severe iron-deficiency anemia (hemoglobin nadir 83 g/L) was managed with iron supplementation.

On October 15, the patient's temperature rose to 39 °C. A second MDT discussion concluded that the persistent fever was attributable to poorly controlled tuberculosis. The anti-tuberculosis regimen was therefore escalated on the same day to isoniazid (0.6 g once daily), rifampicin (0.45 g once daily), linezolid (0.6 g once daily), and moxifloxacin (250 ml once daily). Ethambutol was discontinued due to its increased risk of fetal retrobulbar optic neuritis when combined with linezolid. This intensified regimen was continued until delivery on November 19. In addition, given active hepatitis B virus replication, tenofovir disoproxil fumarate (0.3 g once daily) was initiated on October 15 for antiviral therapy. Written informed consent was obtained after full disclosure of potential adverse effects to the patient and her family.

### Readmission and cesarean delivery (November 11–19, 2025)

On November 11, the patient was readmitted to the Department of Obstetrics and Gynecology at Qinghai Provincial Fourth People's Hospital with complaints of intermittent headache and vomiting for one week, accompanied by fever for one day. Lumbar puncture on November 14 revealed an intracranial pressure of 130 mmH_2_O, and ultrasonography showed an amniotic fluid index of 71 mm with the largest amniotic fluid pocket of 26 mm. Serial prenatal sonographic measurements after admission are shown in Table 2. At the family's request, a lower segment cesarean section was performed on November 19 at 36 weeks of gestation, delivering a live female infant in cephalic presentation, with a birth weight of 2320 g and a 1-min Apgar score of 8.

**Table 2:** Serial prenatal sonographic measurements

Parameter	14 Oct 2025	18 Oct 2025	21 Oct 2025	11 Nov 2025
BPD (mm)	77	78	77	86
AFL (mm)	58	60	58	71
AC (mm)	267	-	-	307
Amniotic fluid(cm)	MVP: 67	AFI: 132	AFI:103, MVP: 37	AFI:71, MVP: 26
FHR (bpm)	163	155	152	167
Placental location	Anterior	Anterior	Anterior	Anterior
Placental grade	0	I	I	II
US-EGA	30w5d	31w2d	32w4d	35w3d
Interpretation	IUP (Cephalic)	IUP (Cephalic)	Viable IUP	Viable IUP

*AC* abdominal circumference, *AFI* amniotic fluid index, *BPD* biparietal diameter, *EGA* estimated gestational age, *FHR* fetal heart rate; *FL* femur length; *IUP* intrauterine pregnancy, *MVP* maximal vertical pocket, *US* ultrasound, *bpm* beats per minute, *w* weeks, *d* days, - means no data

### Maternal and neonatal outcomes

The preterm infant received hepatitis B vaccine and hepatitis B immunoglobulin immediately after birth and was subsequently transferred to Qinghai Provincial Maternal and Child Health Hospital for further observation. Both mother and infant were discharged without complications on December 1, 2025. At the last follow-up on July 30, 2026 (7 months postpartum), the mother remained clinically stable with no evidence of tuberculosis relapse and normal liver and renal function, and the infant demonstrated age-appropriate growth and developmental milestones.

## Discussion

Tuberculosis is a systemic infectious disease capable of affecting multiple organ systems throughout the body, including the central nervous system. Among extrapulmonary manifestations, TBM accounts for approximately 6% of cases and is widely recognized as one of the most devastating forms of tuberculosis, characterized by high case-fatality rates and a substantial risk of permanent neurological impairment [11]. A recent systematic review of pregnancy-related TBM found that among cases with known outcomes, maternal mortality was 23% and fetal/neonatal mortality was 30%; among maternal survivors, 30% had residual neurologic deficits. Notably, diagnosis was missed or delayed in 33% of cases [12].The estimated incidence of active tuberculosis during pregnancy ranges from 1 to 2% [13]. However, clinical diagnosis remains challenging in herder from a pastoral community in Qinghai, particularly in areas with limited medical resources, where diagnostic tools and specialized expertise are often scarce, making the identification of tuberculosis during pregnancy especially difficult.

Pregnancy itself is increasingly recognized as a potential risk factor for tuberculosis. Accumulating evidence indicates that the heightened vulnerability to tuberculosis during gestation may be attributable to physiological immune modulation that occurs in pregnancy: to prevent fetal immune rejection, pregnancy induces partial suppression of type 1 helper T cell (Th1)-mediated cellular immunity while simultaneously promoting type 2 helper T cell (Th2)-mediated humoral immune responses. Since the body's defense against MTB primarily relies on cellular immunity, and active tuberculosis is associated with a pronounced Th2 immune response, this immunological shift may compromise the host's ability to control mycobacterial infection, thereby increasing the risk of tuberculosis reactivation or progression [14]. Timely detection and appropriate treatment are crucial for minimizing the morbidity and mortality associated with TBM. The definitive diagnosis of this disease requires a comprehensive evaluation of clinical manifestations, the epidemiological characteristics of the patient's region of residence, imaging findings, and laboratory test results [15, 16].

MDT decision-making approach: This case illustrates a phase-specific multidisciplinary approach to managing a critically ill pregnant patient with concurrent disseminated tuberculosis, tuberculous meningitis, and hepatitis B. Based on our experience, the MDT interventions can be categorized into four key decision-making domains, each requiring coordinated input from different specialties:

Domain 1: Initial anti-tuberculosis regimen selection and intracranial pressure management.

During the third trimester of pregnancy, the application of conventional osmotic agents for reducing intracranial pressure (including mannitol and glycerol fructose) was limited because of fetal safety concerns. Lumbar cisternal cerebrospinal fluid drainage was prioritized as the preferred intervention for intracranial pressure control in this case. In terms of anti-tuberculosis treatment, the initial regimen consisted of a three-drug combination of isoniazid, rifampicin, and ethambutol. This choice was informed by a balanced consideration of therapeutic efficacy, potential hepatotoxicity, and gastrointestinal tolerability. Pyrazinamide was deliberately omitted from the initial regimen owing to the patient's severe gastrointestinal intolerance to oral medications. Although the World Health Organization recommends a four-drug first-line regimen (rifampicin, isoniazid, ethambutol, and pyrazinamide) for active tuberculosis during pregnancy [17], clinicians should be aware that rifampicin, isoniazid, and pyrazinamide are all associated with hepatotoxic effects, necessitating close monitoring of liver function during treatment.

Domain 2: Regimen escalation in response to clinical deterioration.

When fever (39 °C) and cerebrospinal fluid abnormalities persisted, the MDT re-evaluated the risk–benefit balance. Isoniazid concentrations in CSF approach equilibrium with plasma [18]. Oxazolidinone antibiotics such as linezolid demonstrate good penetration into cerebrospinal fluid, though they carry a higher risk of bone marrow suppression [19, 20]. The CSF permeability of fluoroquinolone such as moxifloxacin has also been confirmed [20]. Ethambutol can partially penetrate the blood–brain barrier under inflammatory conditions but requires caution regarding optic neuritis [21, 22]. Although safety data in pregnant patients remain scarce, the severity of the infection warranted the administration of linezolid and moxifloxacin. Ethambutol was subsequently ceased to minimize the additive risk of fetal optic nerve damage in combination with linezolid. This decision aligned with WHO 2025 guidance, which allows linezolid-containing regimens in pregnant patients under close monitoring [17].

Domain 3: Hepatitis B co-infection management.

Active HBV replication (1.94 × 10^8^ IU/ml) with triple-positive serology posed dual challenges: hepatotoxicity from anti-TB drugs and risk of HBV reactivation. The MDT initiated tenofovir disoproxil fumarate at a dose of 0.3 g daily, the WHO-recommended first-line agent during pregnancy, alongside serial liver function monitoring coordinated jointly by hepatology and clinical pharmacy. The 2026 Chinese Expert Consensus on Screening, Diagnosis, and Treatment of Tuberculosis in Pregnancy emphasizes that management of tuberculosis during pregnancy requires multidisciplinary collaboration involving TB specialists, obstetricians, infectious disease physicians, pharmacists, and neonatologists[13].

Domain 4: Perinatal Coordination.

The MDT determined that delivery could be considered after 34 weeks of gestation, weighing the risks of preterm birth against maternal tuberculosis control. Antenatal fetal monitoring, neonatal hepatitis B immunoprophylaxis, and postpartum follow-up were jointly managed by the departments of obstetrics and gynecology and neonatology. The successful cesarean section and neonatal management reflected the integrated collaboration of the MDT.

A key feature of this MDT model was real-time, cross-specialty communication rather than sequential consultations. Unlike single-specialty management, which often leads to fragmented decisions (e.g., prioritizing infection control without fetal consideration, or delaying aggressive therapy due to pregnancy concerns), the MDT integrated multiple perspectives into a unified, evolving strategy. This approach minimized conflicting recommendations and ensured that critical maternal interventions were not inappropriately postponed.

The present case shares notable similarities with a previously documented case [23], in which a patient at 36 weeks of gestation presented with sudden-onset seizures and was subsequently diagnosed with central nervous system tuberculosis. That patient received a standard four-drug anti-tuberculosis regimen (2HRZE/4 HR) was employed, together with levetiracetam for seizure control. Although mild atonic postpartum hemorrhage complicated the delivery process, prompt and active management led to favorable maternal and neonatal outcomes. The successful resolution of that case likewise relied on close multidisciplinary collaboration, with coordinated input from obstetrics, neurology, pulmonology, and anesthesiology. In comparison to these reports, our MDT process stands out for its explicit management of concurrent HBV infection and its application in a remote pastoral population with limited healthcare access.

Due to the rarity of cases involving pregnancy complicated by multidrug-resistant tuberculosis worldwide, there is currently a lack of high-quality data from randomized controlled trials, cohort studies, or case–control studies. Therefore, this paper presents the application of a multidisciplinary collaboration approach in the diagnosis and treatment of critically ill pregnant patients through a case report format. According to the 2022 meta-analysis published in China Pharmacy titled "Adverse Effects of Drug-Resistant Tuberculosis Treatment on Pregnant Women and Fetuses", exposure to drug-resistant tuberculosis treatment during pregnancy may lead to adverse outcomes such as maternal mortality, miscarriage, neonatal death, preterm birth, low birth weight, developmental delay, and stillbirth. However, the study did not observe any birth defects in newborns. These adverse outcomes may be associated with drug-resistant tuberculosis itself and the severity of the disease. It is therefore crucial to achieve early detection, diagnosis, and treatment of tuberculosis during pregnancy.

Several clinically relevant lessons can be drawn from this case. First, the successful outcome illustrates that early recognition and multidisciplinary collaboration are essential in managing critically ill pregnant patients with concurrent disseminated tuberculosis, tuberculous meningitis, and hepatitis B coinfection. Second, although cranial magnetic resonance imaging was not performed due to the patient's critical condition and late pregnancy, the combination of clinical presentation, cerebrospinal fluid analysis, and chest CT findings was sufficient to guide timely diagnosis and therapeutic decision-making. Third, the patient's herder from a pastoral community in Qinghai background—characterized by high mobility and limited healthcare access—likely contributed to diagnostic delay, highlighting the need for targeted public health strategies to improve tuberculosis awareness and diagnostic capacity in remote pastoral populations. Fourth, this case provides valuable practical insights into the application of a phase-specific MDT approach in a resource-limited setting, demonstrating that coordinated multidisciplinary care can be effectively implemented even in underserved regions. Finally, consistent with the World Health Organization's observation that evidence gaps concerning special populations, including pregnant women, remain unaddressed in current tuberculosis treatment guidelines, this case underscores the urgent need for future research to establish standardized MDT protocols and generate high-quality evidence for tuberculosis management in pregnancy.

## Conclusions

This case demonstrates that an integrated, multidisciplinary strategy—encompassing dynamic, phase-specific decision-making across critical care, respiratory medicine, obstetrics, hepatology, endocrinology, and clinical pharmacy—is essential for optimizing outcomes in pregnant patients with severe disseminated tuberculosis and hepatitis B co-infection. The favorable maternal and neonatal outcomes achieved in a resource-limited pastoral setting further support the feasibility and value of this approach. Future research should focus on establishing standardized MDT protocols for tuberculosis in pregnancy and addressing the evidence gaps concerning special populations identified in current guidelines.

## Data Availability

All data supporting the findings of this study are available within the paper and its Supplementary Information. Chest CT images of the critically ill pregnant patient (Miliary Tuberculosis) are provided in Fig. 1, dynamic changes in cerebrospinal fluid parameters during treatment in a pregnant patient with miliary tuberculosis and tuberculous meningitis are provided in Table 1, serial prenatal sonographic measurements are provided in Table 2.

## Abbreviations

Apgar: Appearance, Pulse, Grimace, Activity, Respiration
CSF: Cerebrospinal fluid
HBV DNA: Hepatitis B virus deoxyribonucleic acid
FDA Category C: FDA Pregnancy Category C
HBeAg: Hepatitis B e antigen
HBsAg: Hepatitis B surface antigen
HBV: Hepatitis B virus
ICP: Intracranial pressure
MDT: Multidisciplinary team
MP: Mycoplasma pneumoniae
MTB: *Mycobacterium tuberculosis*
TBM: Tuberculous meningitis
Th1: Type 1 helper T cell
Th2: Type 2 helper T cell
TSH: Thyroid-stimulating hormone
WHO: World Health Organization
